# A rare case of intact rudimentary horn pregnancy presenting as hemoperitoneum

**DOI:** 10.4103/0974-1208.69335

**Published:** 2010

**Authors:** Ruchi Jain, Neha Gami, Manju Puri, SS Trivedi

**Affiliations:** Department of Obstetrics and Gynecology, Lady Hardinge Medical College, Delhi, India

**Keywords:** Hemoperitoneum, placenta percreta, rudimentary horn pregnancy

## Abstract

The availability of technological advances like ultrasonography (USG) and magnetic resonance imaging (MRI) has made the diagnosis of rudimentary horn pregnancy possible at an early gestation. However, in advanced pregnancy, such cases can sometimes pose a diagnostic dilemma and are recognized only when patient presents with abdominal pain and collapse and is taken for laparotomy. We report one such rare case of a nulliparous female who was carrying on well with her pregnancy till she developed symptoms of acute abdomen at 28 weeks of gestation. She underwent USG and MRI but it was only after laparotomy that a final diagnosis of a pregnancy in a rudimentary horn with placenta percreta perforating through the fundus could be made. There was a significant amount of hemoperitoneum; however, the horn was intact and the fetus could be salvaged. We excised the rudimentary horn with ipsilateral tube and ovary. Post operatively, both the mother and the baby were discharged in healthy condition.

## INTRODUCTION

Pregnancy in a non-communicating uterine horn culminating in the delivery of a live fetus is a rare case. These pregnancies hardly reach viability and often result in rupture of the horn in second trimester. In our case though the patient presented with signs of hemoperitoneum at seven months of pregnancy, the cause was placenta percreta perforating the fundus of an intact horn and timely laparotomy saved both mother and fetus.

## CASE REPORT

A 21-year-old nulliparous lady was referred to our emergency by a general practitioner at 28 weeks of gestation with history of abdominal pain since one week. There was no history of any discharge per vaginum, loss of fetal movements, abdominal trauma, or any bladder or bowel disturbance.

She was gravida two with one spontaneous abortion at 3 months of gestation about one year back, for which a curettage was done. Rest of her medical and surgical history was unremarkable. Her present pregnancy had been uneventful till now and her early pregnancy scans were reported to be normal.

On admission, her general condition was poor, and there were signs of hypotension and tachycardia. Her upper abdomen was soft and non tender while there was guarding and tenderness in lower abdomen. Her uterus was 26 weeks gravid, with contour well made out. Fetal parts were made out with difficulty. The flanks were dull on percussion. Fetal heart sounds could not be auscultated. On per speculum examination, there was no bleeding. Her vaginal examination revealed a soft cervix with os closed, and a firm round nontender 4×4cm mass in the anterior fornix toward the right side of cervix.

**Figure 1:**
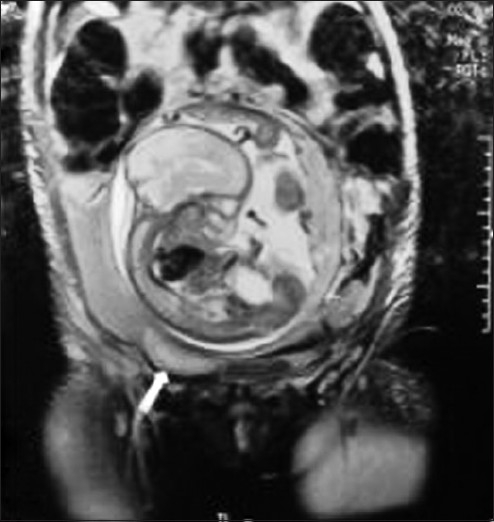
MRI picture showing right uterine horn (arrow) and rudimentary horn above with fetus *in situ*

The patient had undergone an magnetic resonance imaging (MRI) four days back as advised by the treating physician before presenting to this hospital. The MRI reported a didelphys uterus with a single fetus in left uterine body. The placenta was left lateral and there was no free fluid in abdomen [Figure 1]. It also reported the absence of left maternal kidney. Her blood reports at the time of admission showed mild anemia and normal platelet count. Ultrasound was done which showed a bicornuate uterus with a normal right horn and a live 26-27 weeks fetus in the left horn. The placenta was fundoanterior and myometrial continuity was well maintained all around. Free fluid was present in the abdomen which on paracentesis was confirmed to be blood. She was immediately shifted for exploratory laparotomy with a provisional diagnosis of pregnancy in a rudimentary horn with rupture of the horn.

**Figure 2:**
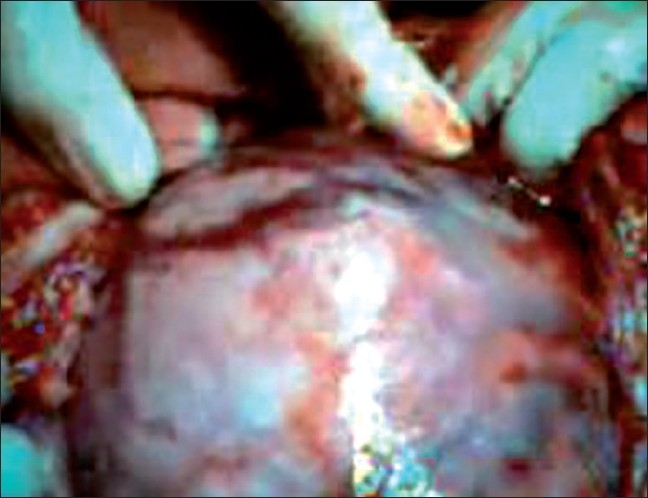
Placental blood vessels seen on the fundus of rudimentary horn

**Figure 3:**
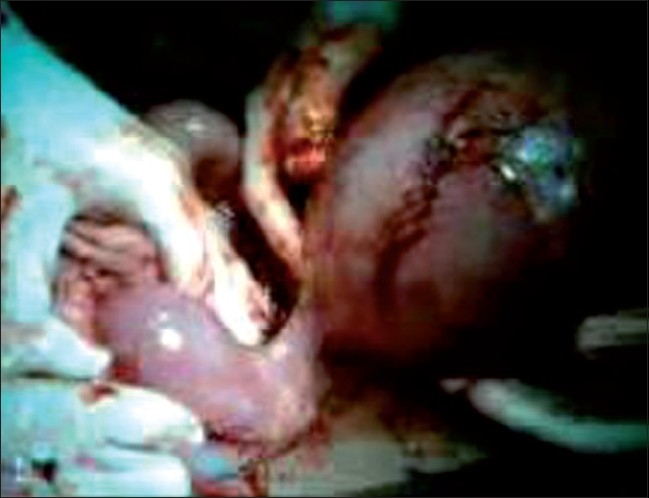
Gravid left rudimentary horn seen attached to unicornuate uterus by a fibrous band

Preoperatively, there was hemoperitoneum of about 1.51. There was an enlarged gravid intact rudimentary horn and brisk bleeding was seen from the prominent blood vessels scattered all over its fundus [Figure 2]. The horn was connected to the left wall of the uterus just above the cervix by a thick fibrous band and the ipsilateral tube and ovary were stretched over the horn [Figure 3]. The fallopian tube and ovary of the right side were healthy. A live 950 g male fetus was extracted from the horn and handed over to the pediatrician. The rudimentary horn and ipsilateral tube and ovary were removed. Left sided kidney and ureter were found to be absent. Abdominal cavity was washed with saline and closed. Patient received two units of packed cells during the operation.

Her postoperative course was uneventful. Pathological evaluation of the specimen confirmed the presence of placenta percreta invading the serosal layer. Microscopic examination confirmed the lack of any communication in the fibrous band connecting the rudimentary horn with the uterus. The neonate was discharged from the hospital after one and a half months in a healthy condition after gaining weight up to 1400 g.

## DISCUSSION

Pregnancy in a noncommunicating rudimentary horn has a reported incidence of 1 in 1,00,000 to 1 in 1,40,000.[1] It occurs following transperitoneal migration of sperm or of fertilized ovum (zygote).[2] It is extremely uncommon for such cases to result in a viable fetus as they often result in rupture of the horn before third trimester. Only 10% cases reach term and the fetal salvage rate is only 2%.[3,4] Rupture occurs commonly because of underdevelopment, variable thickness and poor distensibility of myometrium and dysfunctional endometrium.

As rudimentary horn pregnancies are always associated with catastrophic outcome, every effort should be made to diagnose them at an early gestation. A detailed history should be taken in every patient on her first visit including any complaints of severe dysmenorrhea. However, the rudimentary horn may be underdeveloped and its endometrium non functional and dysmenorrhoea may be absent as seen in our case. A careful pelvic examination in the first trimester showing deviated uterus with a palpable adnexal mass should arouse suspicion of a mullerian anomaly. It can be confirmed by an ultrasound examination though the sensitivity remains only 26%.[5] The enlarging horn with thinned myometrium can obscure the adjacent anatomic structures and the sensitivity further decreases as the gestation increases.

Rudimentary horn pregnancy can be further complicated by placenta percreta due to the poorly developed musculature, scant decidualization and small size of the horn; the reported incidence being 11.9%.[6] MRI has proven to be a very useful tool for the diagnosis of pregnancy with a mullerian anomaly and to confirm the presence of placenta percreta.[7] In our case, the patient’s earlier scans missed the diagnosis and only after she became symptomatic a detailed USG and an MRI revealed the presence of a bicornuate uterus with pregnancy in the left cornu. Even then, the placental invasion remained elusive and was diagnosed only at laparotomy.

Thus we conclude that high clinical suspicion, early diagnosis and timely laparotomy can reduce the perinatal mortality for both mother and fetus. When diagnosed early, excision of rudimentary horn with ipsilateral salpingectomy is the recommended surgical treatment and provides the best prognosis.
